# Seriously misleading results using inverse of Freeman‐Tukey double arcsine transformation in meta‐analysis of single proportions

**DOI:** 10.1002/jrsm.1348

**Published:** 2019-04-23

**Authors:** Guido Schwarzer, Hiam Chemaitelly, Laith J. Abu‐Raddad, Gerta Rücker

**Affiliations:** ^1^ Institute of Medical Biometry and Statistics, Faculty of Medicine and Medical Center University of Freiburg Freiburg im Breisgau Germany; ^2^ Infectious Disease Epidemiology Group, Weill Cornell Medicine‐Qatar Cornell University, Qatar Foundation, Education City Doha Qatar; ^3^ Department of Healthcare Policy & Research, Weill Cornell Medicine Cornell University New York New York USA

**Keywords:** back‐transformation, generalized linear mixed model, harmonic mean, random intercept logistic regression, variance stabilization

## Abstract

Standard generic inverse variance methods for the combination of single proportions are based on transformed proportions using the logit, arcsine, and Freeman‐Tukey double arcsine transformations. Generalized linear mixed models are another more elaborate approach. Irrespective of the approach, meta‐analysis results are typically back‐transformed to the original scale in order to ease interpretation. Whereas the back‐transformation of meta‐analysis results is straightforward for most transformations, this is not the case for the Freeman‐Tukey double arcsine transformation, albeit possible.

In this case study with five studies, we demonstrate how seriously misleading the back‐transformation of the Freeman‐Tukey double arcsine transformation can be. We conclude that this transformation should only be used with special caution for the meta‐analysis of single proportions due to potential problems with the back‐transformation. Generalized linear mixed models seem to be a promising alternative.

## 1 INTRODUCTION

A key application of meta‐analytical methods is the pooling of proportions, such as prevalence of a specific infection or disease.(1, 2, 3, 4) Classic fixed‐effect and random‐effects meta‐analysis methods^5^ are typically used to combine single proportions. In order to use these methods, proportions are generally transformed using either the log,^6^ logit,^7^ arcsine,^8^ or Freeman‐Tukey double arcsine^9^ transformations. These transformations are implemented for pure mathematical reasons, eg,  variance stabilization (details on the transformations are given in Appendix A and summarized in Table A1). For pooling, the transformed proportions and corresponding standard errors are used in the generic inverse variance method.^5^ An alternative yet more elaborate approach based on the logit transformation are generalized linear mixed models (GLMMs),^10^ which account for the binomial structure of the data and thus avoid the generic inverse variance method. Irrespective of the meta‐analysis method and transformation, results are usually presented on the original probability scale after using the corresponding back‐transformation.

Whereas the back‐transformation of meta‐analysis results is straightforward for the log, logit, and arcsine transformations, this is not the case for the Freeman‐Tukey double arcsine transformation, albeit possible.^11^ In order to calculate the inverse of the Freeman‐Tukey double arcsine transformation, a single sample size has to be specified. Accordingly, for a single study, a one to one relation exists between transformation and its inverse, however, in a meta‐analysis with different sample sizes the value of the back‐transformation depends on the specified sample size. Typically, the harmonic mean of sample sizes is used in the back‐transformation.^11^

## 2 CASE STUDY: META‐ANALYSIS ON PREVALENCE OF HEPATITIC C VIRUS INFECTIONS

We report results of meta‐analyses with five studies estimating the prevalence of hepatitis C virus (HCV) infections in the general population of Nepal, which constitute a subset of an unpublished dataset with 28 studies.^12^ This unpublished dataset comprises testing for a total of 972 123 individuals among whom 3696 were HCV antibody positive. The prevalence across studies ranged from 0% to 18.4% with a median of 0.5%. We restrict ourselves to the five‐study subset for didactic reasons; the same issues encountered in this subset also exist in the full dataset.

We conducted classic meta‐analyses using the arcsine, Freeman‐Tukey double arcsine, and logit transformations, respectively. Furthermore, we fitted GLMMs implicitly using the logit transformation. Details on the statistical methods are provided in Appendix A. We used R function metaprop() from R package **meta** 13 (see Supporting Information). Results are summarized in Table 1.

**Table 1:** Estimates and 95% confidence intervals of HCV prevalence meta‐analyses using arcsine, Freeman‐Tukey double arcsine, and logit transformations, respectively

Transformation	Transformed	HCV Infections
(Meta‐analysis Model)	Proportion	per 1000 Observations
Arcsine (fixed)	0.044 (0.042 to 0.046)	1.94 (1.77 to 2.13)
Double arcsine (fixed)	0.044 (0.042 to 0.046)	0.00 (0.00 to 0.00)
Logit (fixed)	−6.231 (−6.323 to −6.139)	1.96 (1.79 to 2.15)
GLMM (fixed)	−6.238 (−6.330 to −6.147)	1.95 (1.78 to 2.14)
Arcsine (random, $\hat{\tau }=0.0003$)	0.044 (0.042 to 0.046)	1.94 (1.76 to 2.13)
Double arcsine (random, $\hat{\tau }=0.0020$)	0.044 (0.041 to 0.048)	0.00 (0.00 to 0.00)
Logit (random, $\hat{\tau }=1.1758$)	−5.451 (−6.649 to −4.254)	4.27 (1.29 to 14.01)
GLMM (random, $\hat{\tau }=0.0000$)	−6.238 (−6.330 to −6.147)	1.95 (1.78 to 2.14)

*Note.* GLMM (fixed) = logistic regression; GLMM (random) = random intercept logistic regression; between‐study variance estimate ${\hat{\tau }}^{2}$.

Abbreviations: GLMM, generalized linear mixed model; HCV, hepatitis C virus.

 

Under the fixed‐effect model, results depicted as transformed proportions (middle column in Table 1) are very similar for the two methods using the arcsine and logit transformations, respectively. Whereas the random‐effects estimates are also very similar with a slightly smaller confidence interval for the arcsine transformation, the results for the two logit methods are rather different due to a very different estimate for the between‐study variance.

For easier interpretation, results are back‐transformed to the original scale. Due to the small prevalences, we express results as HCV infections per 1000 observations. In Table 1 (right column), the results using the inverse of the Freeman‐Tukey double arcsine transformation based on the harmonic mean of 85 are highly irregular with HCV prevalences and confidence limits exactly equal to zero. Under the fixed‐effect model, all of the other three methods show very similar results. Conversely, under the random‐effects model, results for the classic meta‐analysis method using the logit transformation are very different from the other results.

**Figure 1:**
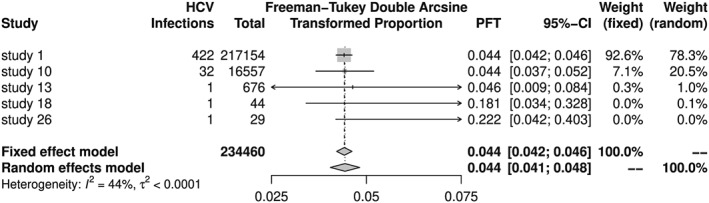
Forest plot of hepatitis C virus (HCV) meta‐analysis with Freeman‐Tukey double arcsine transformation and without back‐transformation of results. PFT, Freeman‐Tukey double arcsine transformed proportion

**Figure 2:**
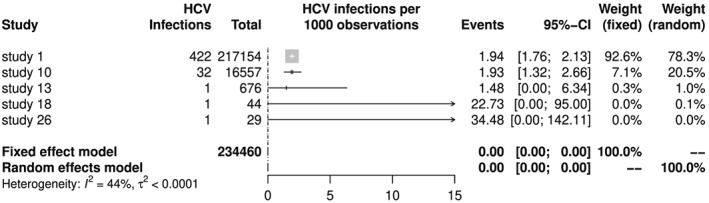
Forest plot of hepatitis C virus (HCV) meta‐analysis with Freeman‐Tukey double arcsine transformation and back‐transformation according to Miller^11^

Looking at Figure 1, we see that the meta‐analysis estimators are reasonable summaries of transformed prevalences. On the other hand, back‐transformed meta‐analysis results are clearly off the mark in Figure 2 with meta‐analysis estimators smaller than all individual study results. Note that the back‐transformation works as expected for individual study results, eg, the prevalence is 1/29 = 0.03448 for study 26, which corresponds to 34.48 HCV infections per 1000 observations.

**Figure 3:**
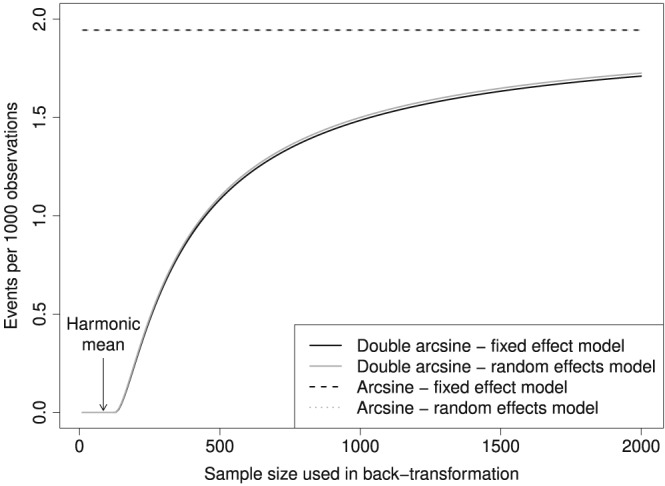
Influence of sample size on results of hepatitis C virus (HCV) meta‐analysis using inverse of Freeman‐Tukey double arcsine transformation according to Miller^11^

The harmonic mean of 85 is obviously the wrong choice in this meta‐analysis with sample sizes ranging from 29 to more than 200 000. Figure 3 shows the influence of sample size on meta‐analysis results (see also Table A2). For sample sizes between 10 and around 120, results are exactly zero for the back‐transformation of the Freeman‐Tukey double arcsine transformation. The number of HCV infections per 1000 observations then steeply increases up to a sample size of 500 when the effect of sample size starts to slowly level out.

**Figure 4:**
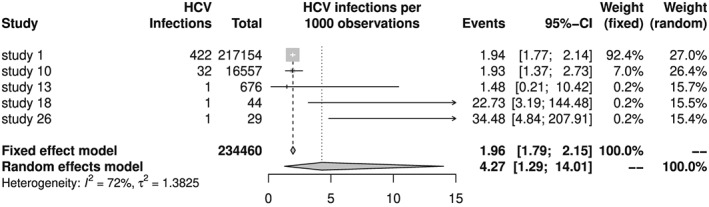
Forest plot of hepatitis C virus (HCV) meta‐analysis using classic method and logit transformation. Confidence intervals for individual studies are based on normal approximation for logit transformed proportions

**Figure 5:**
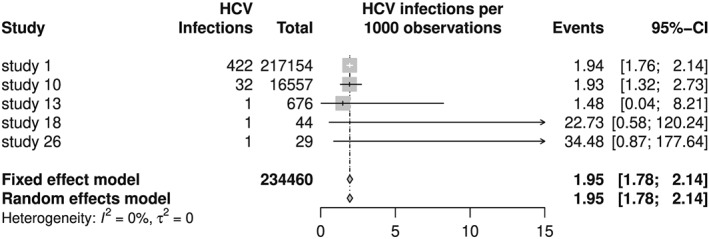
Forest plot of hepatitis C virus (HCV) meta‐analysis using generalized linear mixed model. Confidence intervals for individual studies are based on Clopper‐Pearson method(14, 15)

As noted earlier, the results of the random‐effects model are very different for the two logit methods due to different between‐study variance estimates. This discrepancy can be explained by looking at the confidence intervals of individual studies in the corresponding forest plots (Figures 4 and 5). Confidence intervals, based on the normal approximation, are much narrower for the two smallest studies in the classic random‐effects meta‐analysis (Figure 4) than the confidence intervals, based on the Clopper‐Pearson method taking the binomial distribution into account,(14, 15) in the GLMM meta‐analysis (Figure 5). Apparently, in these two small studies with only 1 HCV infection and less than 50 observations, the assumption of a normally distributed logit transformed proportion is not fulfilled. With increasing numbers of infections and sample sizes, approximate and Clopper‐Pearson confidence intervals get closer to each other. Obviously, the very narrow confidence intervals of the two smallest studies result in an inflated between‐study variance estimate leading to a larger estimate for the pooled mean HCV prevalence and a much wider confidence interval for the pooled mean HCV prevalence.

## 3 DISCUSSION

Our case study shows that meta‐analysis results based on the back‐transformation of the Freeman‐Tukey double arcsine transformation^11^ can be very misleading and even smaller than all individual study results. We observe similar undesirable results in a meta‐analysis using the complete dataset with 28 studies. To our knowledge, this is the first publication reporting such an anomaly and erratic results.

In our view, the main reason for this unexpected behaviour is the very extreme pattern of sample sizes that range from 29 to more than 200 000. The harmonic mean of 85 is much smaller than 3 of the 5 sample sizes. For such highly skewed sample sizes, the harmonic mean is by definition rather small, which may result in nonsensical back‐transformed probabilities.

In order to prevent misleading conclusions for the Freeman‐Tukey double arcsine transformation, several sample sizes could be used to evaluate the sensitivity of meta‐analysis results; however, this may lead to diverging meta‐analysis estimates. In our example, using the arithmetic or geometric mean in the back‐transformation (see Table A2) would result in random‐effects estimates of 1.96 and 1.59 HCV infections per 1000 observations, respectively. Here, results for the harmonic mean are obviously wrong; however, it is rather unclear whether to rely on the results for the arithmetic or geometric mean. All other transformations (arcsine, logit, and log) do not have this intrinsic problem in the presentation of meta‐analysis results.

Overall, the arcsine transformation appears to be the best classic method for the meta‐analysis of single proportions. However, as application of GLMMs for meta‐analysis is nowadays straightforward due to its implementation in common software, there is neither a real reason nor a clear advantage for using an approximate method. Accordingly, we support the viewpoint of  previous works,(10, 16, 17, 18)  recommending the use of GLMMs for the meta‐analysis of single proportions. From our perspective, the only disadvantage of a GLMM is that individual study weights are not available, which we consider as a minor drawback; analysts seeing this differently should use the arcsine transformation.

Our recommendation is purportedly in contrast to advice by Barendregt et al^1^ promoting the use of the Freeman‐Tukey double arcsine transformation over the logit transformation. However, this publication only considered these transformations under the classic meta‐analysis model. We agree with Barendregt et al^1^ that the use of the logit transformation is problematic in inverse variance meta‐analyses with small event numbers or sample sizes; this is also visible in our example. These problems with the logit transformation under the classic meta‐analysis do not translate to GLMMs. The classic meta‐analysis model assumes that treatment estimates of individual studies follow a normal distribution that is obviously critical in studies with small numbers of events and observations. The arcsine and Freeman‐Tukey double arcsine transformation are less affected by this normality assumption than the logit transformation. However, GLMMs taking into account the binomial structure of the data are not affected by this problem at all.(10, 16)

## 4 CONCLUSIONS

Our case study shows that the Freeman‐Tukey double arcsine transformation should only be used with special caution for the meta‐analysis of single proportions due to potential problems in the back‐transformation of meta‐analysis results. In our view, a sensitivity analysis using other sample sizes is mandatory for this transformation. GLMMs seem to be a promising alternative which is nowadays available in common meta‐analysis software.

## CONFLICT OF INTEREST

The author reported no conflict of interest.

## Supporting information



Supporting info item

Supporting info item

## APPENDIX A STATISTICAL METHODS

### A.1

We consider a meta‐analysis of *K* studies where each study reports the number of events, *a*_*k*_, and the number of observations *n*_*k*_, *k* = 1,…,*K*. We assume that the number of events follows a binomial distribution. Specifically, cell count *a*_*k*_∼Binomial(*n*_*k*_,*p*_*k*_), where *p*_*k*_ denotes the probability of the event in study *k*. These probabilities are estimated from the observed number of events and sample sizes by ${\hat{p}}_{k}=a_{k}/n_{k}$.

### A.1 Transformations

In this subsection, we briefly introduce the arcsine, Freeman‐Tukey double arcsine, and logit transformations in the context of a single study. In the next subsection, the use of these transformations in meta‐analyses will be described.

#### A.1.1 Arcsine transformation

The arcsine‐transformed event probability $\theta_{k}^{AS}$ 8 is defined as

$$\theta_{k}^{AS}=\arcsin\sqrt{p_{k}}\;.$$

An estimate of $\theta_{k}^{AS}$ is given by replacing *p*_*k*_ with ${\hat{p}}_{k}$. The main advantage of this transformation is the property of variance stabilization. The approximate variance of ${\hat{\theta }}_{k}^{AS}$ is calculated using

$$\hat{\mathrm{Var}}\;({\hat{\theta }}_{k}^{AS})=\frac{1}{4\;n_{k}},$$

where the approximation improves as *n*_*k*_ increases. Notice that the approximate variance of ${\hat{\theta }}_{k}^{AS}$ only depends on the sample size. A confidence interval for $\theta_{k}^{AS}$ can be constructed as

$${\hat{\theta }}_{k}^{AS}\;\pm \;z_{1-\frac{\alpha }{2}}\;\text{S.E.}({\hat{\theta }}_{k}^{AS})$$

with standard error $\text{S.E.}({\hat{\theta }}_{k}^{AS})=\sqrt{\hat{\mathrm{Var}}\;({\hat{\theta }}_{k}^{AS})}$ and $z_{1-\frac{\alpha }{2}}$ denoting the $1-\frac{\alpha }{2}$ quantile of the standard normal distribution.

#### A.1.2 Freeman‐Tukey double arcsine transformation

The Freeman‐Tukey double arcsine‐transformed event probability $\theta_{k}^{FT}$ 9 is an average of two arcsine‐transformed probabilities. Its estimate is given by

$${\hat{\theta }}_{k}^{FT}=0.5\left(\arcsin\sqrt{\frac{a_{k}}{n_{k}+1}}+\arcsin\sqrt{\frac{a_{k}+1}{n_{k}+1}}\right).$$

The Freeman‐Tukey double arcsine transformation was introduced in order to improve on the variance stabilizing property of the arcsine transformation. The approximate variance of ${\hat{\theta }}_{k}^{FT}$ is

$$\hat{\mathrm{Var}}\;({\hat{\theta }}_{k}^{FT})=\frac{1}{4\;n_{k}+2},$$

where the approximation—again—improves as *n*_*k*_ increases. A confidence interval for $\theta_{k}^{FT}$ can be constructed following the same methodology for that of the arcsine transformed probability described above.

#### A.1.3 Logit transformation

The logit transformation is another classic transformation^7^ defined as

$$\theta_{k}^{LO}=\log\left(\frac{p_{k}}{1-p_{k}}\right)\;.$$

Again, an estimate of $\theta_{k}^{LO}$ is given by replacing *p*_*k*_ with ${\hat{p}}_{k}$. The approximate variance of ${\hat{\theta }}_{k}^{LO}$ is

$$\hat{\mathrm{Var}}\;({\hat{\theta }}_{k}^{LO})=\frac{1}{a_{k}}+\frac{1}{n_{k}-a_{k}}\;.$$

It is clear from this variance formula that the approximate variance of a logit transformed proportion can become infinite if the number of events is zero or equal to the sample size. Typically, in this situation, a small increment is added to each denominator in order to yield a finite variance estimate.

A confidence interval for $\theta_{k}^{LO}$ can be constructed following the same methodology for that of the arcsine transformed probability described earlier.

### A.2 Meta‐analysis of single proportions

We briefly describe both the classic meta‐analysis method assuming approximate normally distributed study effects (ie, prevalence measures) as well as the generalized linear mixed model taking the binary structure of the data into account.

All methods are available in R function metaprop() from R package **meta**.^13^

#### A.2.1 Classic random‐effects model

Classic fixed‐effect and random‐effects meta‐analysis methods using the inverse variance method^5^ can be implemented to combine single proportions. As the random‐effects model is a generalization of the fixed‐effect model, we only introduce the random‐effects model, which is defined as

$$\begin{array}{ccc} {\hat{\theta }}_{k}=\; & \theta_{k}+\epsilon_{k},\;\epsilon_{k}\overset{\text{i.i.d.}}{∼}N(0,\sigma_{k}), & \\ \theta_{k}=\; & \theta +u_{k},\;u_{k}\overset{\text{i.i.d.}}{∼}N(0,\tau^{2})\;, \end{array}$$

where the *ϵ*'s and *u*'s are independent. This model contains two sources of variation: the within‐study variances $\sigma_{k}^{2}$, *k* = 1,…,*K*, and the between‐study variance *τ* ^2^. The classic meta‐analysis methods assume that the variances $\sigma_{k}^{2}$ are estimated without error by ${\hat{\sigma }}_{k}^{2}$. The estimated effects ${\hat{\theta }}_{k}$ and corresponding standard errors *σ*_*k*_ (which are assumed known) are used to estimate *τ* ^2^ with the restricted maximum likelihood method.^19^ Results are very similar using the classic DerSimonian and Laird estimator, which is still the default in most statistical software for meta‐analysis. The fixed‐effect model is a special case when *τ* ^2^ = 0. Accordingly, results of fixed‐effect and random‐effects meta‐analysis are identical if the estimate ${\hat{\tau }}^{2}$ equals zero.

Given estimates $({\hat{\theta }}_{k},{\hat{\sigma }}_{k}),$ the random‐effects estimate of *θ*, denoted by ${\hat{\theta }}_{R}$, is

$${\hat{\theta }}_{R}=\frac{\sum_{k=1}^{K}{\hat{\theta }}_{k}/({\hat{\sigma }}_{k}^{2}+{\hat{\tau }}^{2})}{\sum_{k=1}^{K}1/({\hat{\sigma }}_{k}^{2}+{\hat{\tau }}^{2})}\;,$$

which is a weighted average of the individual effect estimates ${\hat{\theta }}_{k}$ with weights $w_{k}=1/({\hat{\sigma }}_{k}^{2}+{\hat{\tau }}^{2})$.

**Table A1:** Definition and properties of prevalence transformations with number of events a and total sample size n

		Approximate	
Transformation	Estimate	Variance	Comments
log^6^	log(a/n)	$\frac{1}{a}-\frac{1}{n}$	Infinite estimate and variance for zero events
logit^7^	$\log\left(\frac{a/n}{1-a/n}\right)$	$\frac{1}{a}+\frac{1}{n-a}$	Infinite estimate and variance for zero or all events
arcsine^8^	$\arcsin\sqrt{a/n}$	$\frac{1}{4\;n}$	Variance stabilizing; defined for zero events
Double arcsine^9^	$0.5\;\left(\arcsin\sqrt{a/(n+1)}+\right.$	$\frac{1}{4\;n+2}$	Outperforms arcsine for small prevalences;
	$\left.\arcsin\sqrt{\left(a+1)/(n+1\right)}\right)$		sample size needed in back‐transformation^11^

**Table A2:** Estimated number of HCV infections per 1000 observations for additional sample sizes in fixed‐effect and random‐effects meta‐analyses using the back‐transformation of the Freeman‐Tukey double arcsine method

	HCV Infections per 1000 Observations		
Sample Size	Fixed Effect	Random Effects	Mean
85	0.000	0.000	Harmonic
500	1.083	1.097	
1000	1.486	1.500	
1254	1.575	1.590	Geometric
10 000	1.902	1.917	
46 892	1.941	1.956	Arithmetic
100 000	1.947	1.962	
1 000 000	1.951	1.966	

The variance of ${\hat{\theta }}_{R}$ is estimated by

$$\hat{\mathrm{Var}}\;({\hat{\theta }}_{R})=\frac{1}{\sum_{k=1}^{K}w_{k}}$$

and a (1‐*α*) confidence interval for ${\hat{\theta }}_{R}$ can be calculated using

$${\hat{\theta }}_{R}\pm z_{1-\frac{\alpha }{2}}\;\text{S.E.}\;({\hat{\theta }}_{R})$$

with standard error $\text{S.E.}\;({\hat{\theta }}_{R})=\sqrt{\hat{\mathrm{Var}}\;({\hat{\theta }}_{R})}$.

A fixed‐effect meta‐analysis can be conducted by assuming a between‐study variance *τ* ^2^ = 0 resulting in a fixed‐effect estimate ${\hat{\theta }}_{F}$.

Instead of ${\hat{\theta }}_{k}$ and ${\hat{\sigma }}_{k}$, we use ${\hat{\theta }}_{k}^{AS}$ and $\text{S.E.}({\hat{\theta }}_{k}^{AS})$ for the arcsine method, ${\hat{\theta }}_{k}^{FT}$ and $\text{S.E.}({\hat{\theta }}_{k}^{FT})$ for the Freeman‐Tukey double arcsine method, and ${\hat{\theta }}_{k}^{LO}$ and $\text{S.E.}({\hat{\theta }}_{k}^{LO})$ for the logit method. We denote the corresponding fixed‐effect and random‐effects estimates as ${\hat{\theta }}_{F}^{AS}$, ${\hat{\theta }}_{R}^{AS}$, ${\hat{\theta }}_{F}^{FT}$, ${\hat{\theta }}_{R}^{FT}$, ${\hat{\theta }}_{F}^{LO}$, and ${\hat{\theta }}_{R}^{LO}$, respectively.

#### A.2.2 Generalized linear mixed model

An excellent tutorial^10^ describes how generalized linear mixed models can be utilized in the meta‐analysis of event outcomes. One special case considered in the paper is the meta‐analysis of single proportions, which—like the classic meta‐analysis model—assumes a normal distribution for the effect size (ie, transformed proportion) across studies. However, a binomial distribution is assumed for the number of events within a study, ie, $a_{k}∼\text{Binomial}\left(n_{k},p_{k}\right)$. Using the above defined logit transformed proportion $\theta_{k}^{LO}$, this relation can be re‐expressed in the following way to define the random‐effects model

$$\begin{array}{ccc} a_{k} & \overset{\text{i.i.d.}}{∼}\;\text{Binomial}\left(n_{k},\frac{\exp(\theta_{k}^{LO})}{1+\exp(\theta_{k}^{LO})}\right), & \\ \theta_{k}^{LO} & \overset{\text{i.i.d.}}{∼}\;\theta +u_{k}\;u_{k}\overset{\text{i.i.d.}}{∼}N(0,\tau^{2}). \end{array}$$

This model uses the binomial likelihood $\exp{\left(\theta_{k}^{LO}\right)}^{a_{k}}/(1+\exp{\left(\theta_{k}^{LO}\right)}^{n_{k}}$ instead of the likelihood from the normal distribution^10^ and is also known as a random intercept logistic regression model that implicitly uses the logit transformation. Accordingly, the GLMM estimates ${\hat{\theta }}_{F}^{GL}$ and ${\hat{\theta }}_{R}^{GL}$ correspond to the logit transformed probabilities in the fixed‐effect and random‐effects model, respectively.

Estimation of GLMMs for meta‐analysis of single proportions is straightforward with R function metaprop() by specifying argument method = "GLMM".

In principle, individual study weights could be derived from the likelihood contribution of each individual study; however, this information is at the moment not available in the utilized R software. Alternatively, the width of the Clopper‐Pearson confidence intervals that also takes the binomial data structure into account(14, 15) could be used to get approximate study weights.

### A.3 Back‐transformations

For a single study, several statistical methods exist to calculate a confidence interval for a single proportion.(14, 15) These methods do not use the arcsine or the Freeman‐Tukey double arcsine transformations, and therefore, the back‐transformation is not strictly relevant for individual study results. However, in a meta‐analysis context, the back‐transformation of the (double) arcsine as well as the logit transformation is essential to report results on the original scale, ie, as proportions.

#### A.3.1 Arcsine back‐transformation

The back‐transformation/inverse of the arcsine transformation is defined as

$$p_{k}^{AS}=\sin{\left(\theta_{k}^{AS}\right)}^{2}\;.$$

This back‐transformation can be used for a single study as well as the result of a meta‐analysis, eg, for the random‐effects estimate ${\hat{\theta }}_{R}^{AS}$ and its lower and upper confidence limits.

#### A.3.2 Inverse of Freeman‐Tukey double arcsine transformation

Miller^11^ introduced the back‐transformation of the Freeman‐Tukey double arcsine transformation that was published almost 30  years after the initial publication.^9^ For study *k*, the back‐transformation is defined as

$$p_{k}^{FT}=0.5\;\left(1-\text{sgn}(\cos(\theta_{k}^{FT}))\sqrt{1-{\left(\sin(2\;\theta_{k}^{FT})+\left[\sin(2\;\theta_{k}^{FT})-1/\sin(2\;\theta_{k}^{FT})\right]/n_{k}\right)}^{2}}\right)\;.$$

This rather complex back‐transformation arises from using an average of two arcsine transformed proportions. The sample size *n*_*k*_ is included in the back‐transformation, which is no problem for a single study. However, in a meta‐analysis with different sample sizes, a single sample size has to be specified to apply the back‐transformation. Miller^11^ suggested to use the harmonic mean of the sample sizes, ie, $ñ=K/\sum_{k=1}^{K}\frac{1}{n_{k}}$. Accordingly, this harmonic mean ñ and the meta‐analysis estimate ${\hat{\theta }}_{F}^{FT}$ or ${\hat{\theta }}_{R}^{FT}$ are used in the back‐transformation.

#### A.3.3 Inverse of logit transformation

The inverse of the logit transformation is defined as

$$p_{k}^{LO}=\frac{\exp(\theta_{k}^{LO})}{1+\exp(\theta_{k}^{LO})}\;.$$

This well‐known back‐transformation can be used both for a single study and in a meta‐analysis setting (classic method or GLMM).
